# Thermodynamic Characteristics of Oxidation and Combustion of Coal under Lean-Oxygen Conditions

**DOI:** 10.1021/acsomega.1c01096

**Published:** 2021-07-01

**Authors:** Haiyan Wang, Jinglei Li, Xiao Chen, Cheng Fan, Peipei Wang, Lang Hu

**Affiliations:** School of Emergency Management and Safety Engineering, China University of Mining and Technology, Beijing 100083, P. R. China

## Abstract

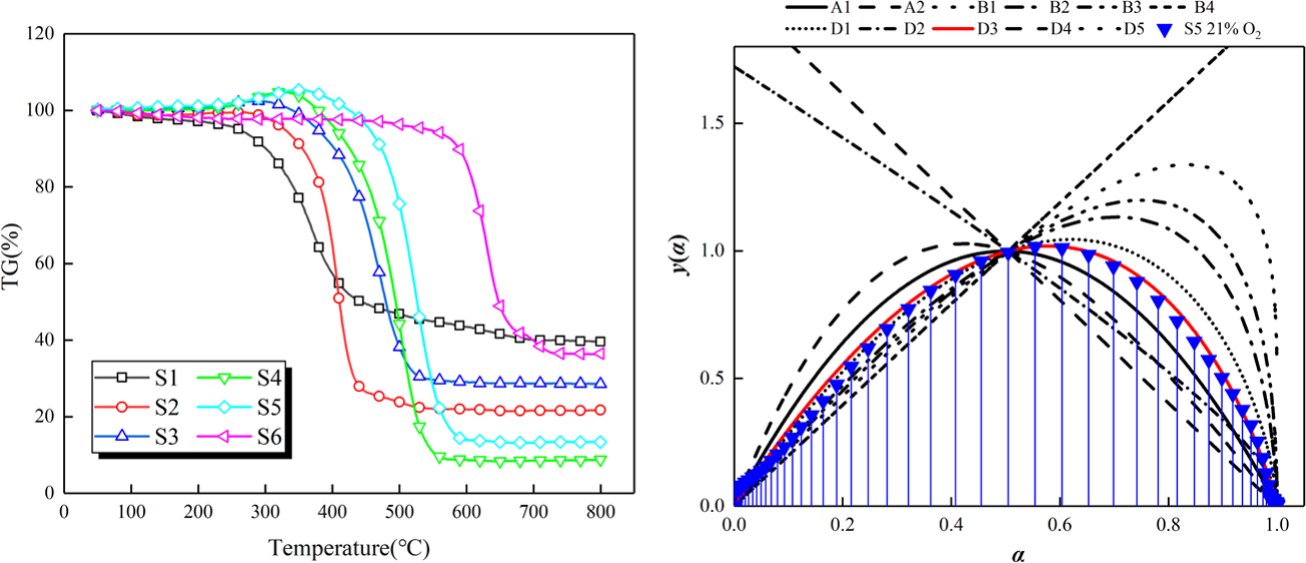

Coal spontaneous combustion (CSC) often occurs in environments that are poorly ventilated or under lean-oxygen environments in coal mines or coal seam outcrops. The understanding of the thermodynamic properties of CSC under lean-oxygen conditions is important to avoid safety and environmental problems. In this paper, the mass variation and critical temperature (the minimum temperature required for coal reaction in each stage) of six coals during CSC under lean-oxygen conditions were investigated using thermogravimetric methods. Furthermore, the thermodynamics parameters and kinetic compensation processes during CSC under lean-oxygen conditions were analyzed. The results showed that the oxidation and combustion of coal under lean-oxygen conditions was affected by the coal rank and oxygen concentration. The increase in the critical temperature was more significant when the oxygen concentration was reduced from 10 to 5%. The mechanism functions most likely to cause CSC at different oxygen concentrations were similar. The reaction mechanisms of high-rank coals at a low-temperature oxidation stage were more influenced by oxygen concentration than low-rank coals. Evaluations of kinetic behavior showed that activation energy decreased in a linear manner as the oxygen concentration decreased. The mathematical relationship between the activation energy and the pre-exponential factor indicated that there was a kinetic compensation process during CSC under lean-oxygen conditions. It is also worth noting that the effect of the coal rank on the thermodynamic characteristics of CSC is better than that of oxygen concentration. This work is helpful for enhancing the prediction and prevention of CSC.

## 1 Introduction

Coal spontaneous combustion (CSC) is a natural objective phenomenon that has existed for millions of years.^1^ CSC is a common occurrence around the world, especially in major coal-producing countries.^2^ Every year, CSC would burn many ecological resources and emit large amounts of toxic and harmful (CO, CO_2_, CH_4_, SO_2_, NO_*X*_, etc.) gases. Half of the global energy industry’s total SO_2_ emissions come from CSC, and CSC accounts for around 20% of global NO_*X*_ emissions.^3^ A series of problems caused by CSC, such as the release of toxic and harmful gases, ground subsidence, and mine production accidents, not only threatens the production activities of human beings but also destroys the ecological environment, even endangering human health.^4,5^ Therefore, the characteristics such as temperature variation,^6−8^ reaction generation,^9−11^ and evolution of functional groups^12,13^ in CSC are of great attention.

Oxygen, as one of the indispensable conditions for CSC, has a significant effect on the oxidation and combustion of coal.^14,15^ In recent years, the effect of oxygen concentration on the combustion characteristics of coal is of great concern. Many scholars have found that the enriched oxygen condition has a significant promotional effect on coal combustion. Based on the study of coal ignition in a high-temperature oxidizer at different oxygen concentrations, Ponzio^16^ found that the time required for the coal ignition varies with the oxygen concentration and oxidizer temperature and that it is more affected by the enriched oxygen condition. The same point was made by Chao,^17^ who argued that the temperature of coal ignition under enriched oxygen conditions decreased with increasing oxygen concentration. By studying the convective effect of bituminous coal ignition under enriched oxygen conditions, Liu^18^ pointed out that nonuniform combustion occurred when coal was burned at an oxygen concentration of 21% and the Reynolds number of 2, 8, and 16. Under enriched oxygen conditions, however, nonuniform combustion occurred only at the Reynolds number of 8. The results indicated that the effect of convection on oxygen diffusion is superior to the sweep air under specific Reynolds number and enriched oxygen volume flow conditions. Qi^19^ found that the difference in kinetic parameters decreased with decreasing oxygen concentration at different stages of coal oxidation. Thus, it can be seen that oxygen-enriched conditions have a positive influence on efficiency and technology improvement of coal combustion. However, most experiments of coal combustion have been conducted under enriched oxygen conditions. In contrast, CSC occurs more often under poorly ventilated or lean-oxygen conditions, particularly in coal mines or coal seam outcrops. It has been suggested that the critical oxygen concentration that caused CSC was between 2 and 18%,^20−22^ and the gas generated under lean-oxygen conditions was more harmful than that under enriched oxygen conditions. Xiao^23^ believed that as the coal particle size was lower than 0.098 mm, the difference in critical temperatures of CSC showed a style of “M” with the increase of oxygen concentration, and the difference reached a maximum when the oxygen concentration was 14.9%. Perdochova^24^ addressed experimentally that the coal ignition temperature decreased with increasing oxygen concentration under lean-oxygen conditions. The oxygen concentration and mechanism of the coal reaction affected the number of gas generations under the condition in which the temperature was 423 K. Moreover, Perdochova also noted that the concentration of gas generations at the oxygen concentration of 21% is higher than those at 15%. Wang^25^ obtained an improved kinetic model that is suitable for describing the process of CSC when the oxygen concentration is 10% through thermogravimetric (TG) methods. By TG experiments, Li^26^ investigated the thermodynamic parameters during CSC under lean-oxygen conditions and pointed out that the values of activation energy fluctuated marginally as the oxygen concentration varied.

In the thermodynamic theory, the activation energy is critical for representing the energy required for coal reaction and has attracted widespread attention. Indeed, the lower the activation energy, the lower the difficulty of CSC. However, the complex molecular structure of coal led to multiple reactions simultaneously. Thus, the activation energy obtained is inaccurate.^14,27^ Due to the complexity of the coal structure, there is no fully applicable method to obtain the kinetic parameters during CSC. The kinetic parameters are often obtained by the kinetic methods that are most suitable for describing the process of CSC, and the methods are gradually improving and gaining acceptance. By the kinetic analysis of coal pyrolysis under nonisothermal conditions, Mianowski^28^ presented the main problems for the calculation of kinetic parameters during coal pyrolysis and obtained a kinetic model that explained the process of CSC more accurately. Kaljuvee^29^ concluded that the activation energy is related to the coal properties. The higher organic matter content of coal indicates higher activation energy. Besides, the ranks and particle size of coal also affect the activation energy.^23,27^ Under different oxygen concentration conditions, the difference in activation energy between the slow oxidation and rapid oxidation stages of coal pyrolysis is large.^19^ Yao^30^ suggested that the mechanism most likely to cause CSC is susceptible to mutation under lean-oxygen conditions. In contrast, the mechanism is relatively stable under enriched oxygen conditions. However, the current research studies on the influence of oxygen concentration on thermodynamic behaviors of CSC are mainly based on a single experiment condition, resulting in the inability to investigate the influencing factors of the kinetic parameters during CSC from multiple perspectives. It is necessary to study the thermodynamic behaviors of CSC under different conditions.

In this paper, six coals with different ranks are used as experimental samples. The mass variation and critical temperatures during CSC are determined under lean-oxygen conditions by TG methods, and the influence of oxygen concentration on the distribution of critical temperature is analyzed. The kinetic parameters during CSC were obtained by the integral method, and the kinetic compensation effect was verified. Therefore, the results complement the deficiencies of previous studies, enhancing the understanding of CSC and aiding in the prevention and control of CSC disasters.

## 2 Experimental Materials, Procedures, and Principle

### 2.1 Materials

The coal samples were collected from the major coal-producing regions in China. Their specific details are listed in Table 1. The samples were collected at the mine site and transported back to the laboratory in sealed sample bags to avoid oxidation in the air. The results of proximate analyses of the coal samples are listed in Table 1.

**Table 1:** Proximate Analyses of Coal Samples

coal sample	number	location	*M*_ad_ (%)	*V*_ad_ (%)	FC_d_ (%)	*A*_d_ (%)
lignite coal	S1	Inner Mongolia Autonomous Region	16	33.5	31.7	34.8
nonstick coal	S2	Inner Mongolia Autonomous Region	12.8	29.9	56.8	13.2
gas coal	S3	Hebei Province	2.9	29.5	44.7	25.8
coking coal	S4	Hebei Province	1.1	24.5	69.1	6.2
lean coal	S5	Shanxi Province	0.8	13.05	76.5	10.4
anthracite coal	S6	Shanxi Province	3.3	7.8	57	35.2

### 2.2 Experimental Equipment and Conditions

The experimental apparatus used was STA449F3 (Netzsch GmbH, Selb, Germany). The apparatus consists of a common TG–differential scanning calorimetry (DSC) mount for simultaneous measurement of thermal effects and mass variation. Its TG resolution is 0.1 μg, and the DSC resolution is less than 1 μw. In addition, the instrument is equipped with solenoid valves for controlling purge gas and protective gas and a mass flow meter for precise control of the gas flow and atmosphere. Before the test, the coal samples were crushed and sieved into particles of size 200 mesh. Around 5 mg of each coal sample was taken for the experiment, and the ventilation flow rate was set to 30 mL/min to approximate the reality of CSC. CSC requires a long process of heat storage. The self-heating and heat production capacity of coal are subject to many factors, such as coal properties, porosity, water content, pyrite content, and the heat storage environment.^31^

From the TG experiments, Li^32^ found that the values of thermodynamic parameters of CSC varied similarly with oxygen concentration (5, 9, 13, 17, and 21%) at different heating rates. Moreover, existing literature reports suggested that the heating rate in the early stage of CSC ranges from 4.2 to 5.6 °C/min.^33−36^ Therefore, the heating rate used in this study was 5 °C/min. Four oxygen concentrations of 5, 10, 15, and 21% were used. The pulverized coal is heated from 50 to 800 °C at each oxygen concentration. During the experiment, the apparatus constantly monitored its thermodynamic data and saved it in real time.

### 2.3 Kinetic Methods

#### 2.3.1 Method of Inferring the Kinetic Model

CSC is an extremely complex process, the kinetic mechanism of which cannot be accurately obtained. The Malek method determines the kinetic model by a function *y*(α), which is given as follows^37^
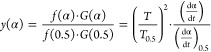
1where *y*(α) is the defined function, α is the conversion rate in %, *T* is the temperature corresponding to α in K, and *T*_0.5_ is the temperature when α is equal to 0.5 in K. *f*(α) and *G*(α) are the differential and integral forms of the mechanistic function, respectively. dα/d*t* is the reaction rate. (dα/d*t*)_0.5_ corresponds to the rate when α = 0.5.

The conversion rate can be calculated using the following equation
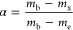
2where *m*_b_ and *m*_e_ are the starting and ending masses of the coal samples, respectively, in milligrams. *m*_x_ is the mass of the coal sample at a given moment, in milligrams.

Theoretical *y*(α) values for various differential and integral kinetic model were determined using the [*f*(α)·G(α)/*f*(0.5)·G(0.5)] term. On the other hand, experimental values were obtained by multiplying the experimental values of (*T*/*T*_0.5_)^2^ and [(dα/d*t*)/(dα/d*t*)_0.5_]. Then, the theoretical and experimental master plots are derived by plotting the experimentally and theoretically measured *y*(α) values as a function of α. The theoretical model with the best fit to the experimental master plot was used as the most suitable model to depict the thermal oxidation process. The differential and integral functions of these theoretical models are detailed in Table 2. In this study, 11 kinetic models were investigated (Table 2).

**Table 2:** Common Kinetic Mechanism Functions

number	function name	mechanism model	*f*(α)	*G*(α)
A1	Mample principle (level II)	random nucleation and subsequent growth	(1 – α)^2^	(1 – α)^−1^ – 1
A2	Mample principle (level III)	random nucleation and subsequent growth	(1 – α)^3^	1/2[(1 – α)^−2^ – 1]
B1	Valensi equation	two-dimensional diffusion	[−In(1 – α)]^−1^	α + (1 – α) In(1 – α)
B2	reaction order	phase boundary reaction, *n* = 1/3	3 (1 – α)^2/3^	1 – (1 – α)^1/3^
B3	reaction order	Phase boundary reaction, *n* = 1/2	2 (1 – α)^1/2^	1 – (1 – α)^1/2^
B4	Parabolic law	one-dimensional diffusion	1/2 α^–1^	α^2^
D1	Jander equation	three-dimensional diffusion	3/2(1 – α)^2/3^[1-(1 – α)^1/2^]^−1^	[1 – (1 – α)^1/3^]^2^
D2	Ginstling–Brountein equation	three-dimensional diffusion	3/2[(1 – α)^−1/3^ – 1]^−1^	1 – 2/3α – (1 – α)^2/3^
D3	Zhuralev–Lesokin–Tempelman equation	three-dimensional diffusion	3/2(1 – α)^4/3^[(1 – α)^−1/3^ – 1]^−1^	[(1 – α)^−1/3^ – 1]^2^
D4	second-order chemical reaction	decelerator curve	(1 – α)^2^	(1 – α)^−1^
D5	zero order	*n* = 0	1	α

#### 2.3.2 Coats–Redfern (CR) Method

The Coats–Redfern method is one of the standard integration methods for obtaining the activation energy, which is as follows^38^
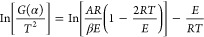
3where *G*(α) is a function, which describes various reaction models in the differential form. dα/d*T* is the first-order derivative of α over *T*. *A*, R, and β are, respectively, the pre-exponential factor (min^–1^), gas constant (8.314 × 10^–3^ kJ/mol k), and heating rate (°C/min), and *E* is performance activation energy in kJ/(mol).

Since the value of (2RT/E) is much less than 1, the  term in eq 3 can be approximately equivalent to In(*AR*/β*E*), and eq 3 can be represented as
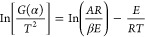
4

A straight line can be obtained from the plot of In[*G*(α)/*T*^2^]and 1/*T*. Here, the *E* and *A* values can be obtained from the slope and intercept of the line, respectively.

#### 2.3.3 Kinetic Compensation Effect

When discussing the relationship between activation energy (*E*) and the reaction rate at a given temperature, generally speaking, the higher activation energy indicates a lower reaction rate. However, this statement holds only if the pre-exponential factor (*A*) is constant. For a multiphase reaction such as CSC, *E* is proportional to *A*. In other words, there is a compensatory relationship between these two kinetic parameters that have opposite effects on the reaction rate, called the “kinetic compensation effect”. It is noteworthy that the kinetic compensation effects are widespread in most systems and reaction processes. Consequently, the kinetic compensation effect can be considered when there is a linear relationship between In *A* and *E*, which can be expressed using eq 5
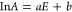
5where *a* and *b* are the compensation factors. Plotting In *A* versus *E*, the *a* and *b* values would be obtained by the slope and intercept of the line, respectively.

## 3 Results and Discussion

### 3.1 TG Analysis

**Figure 1:**
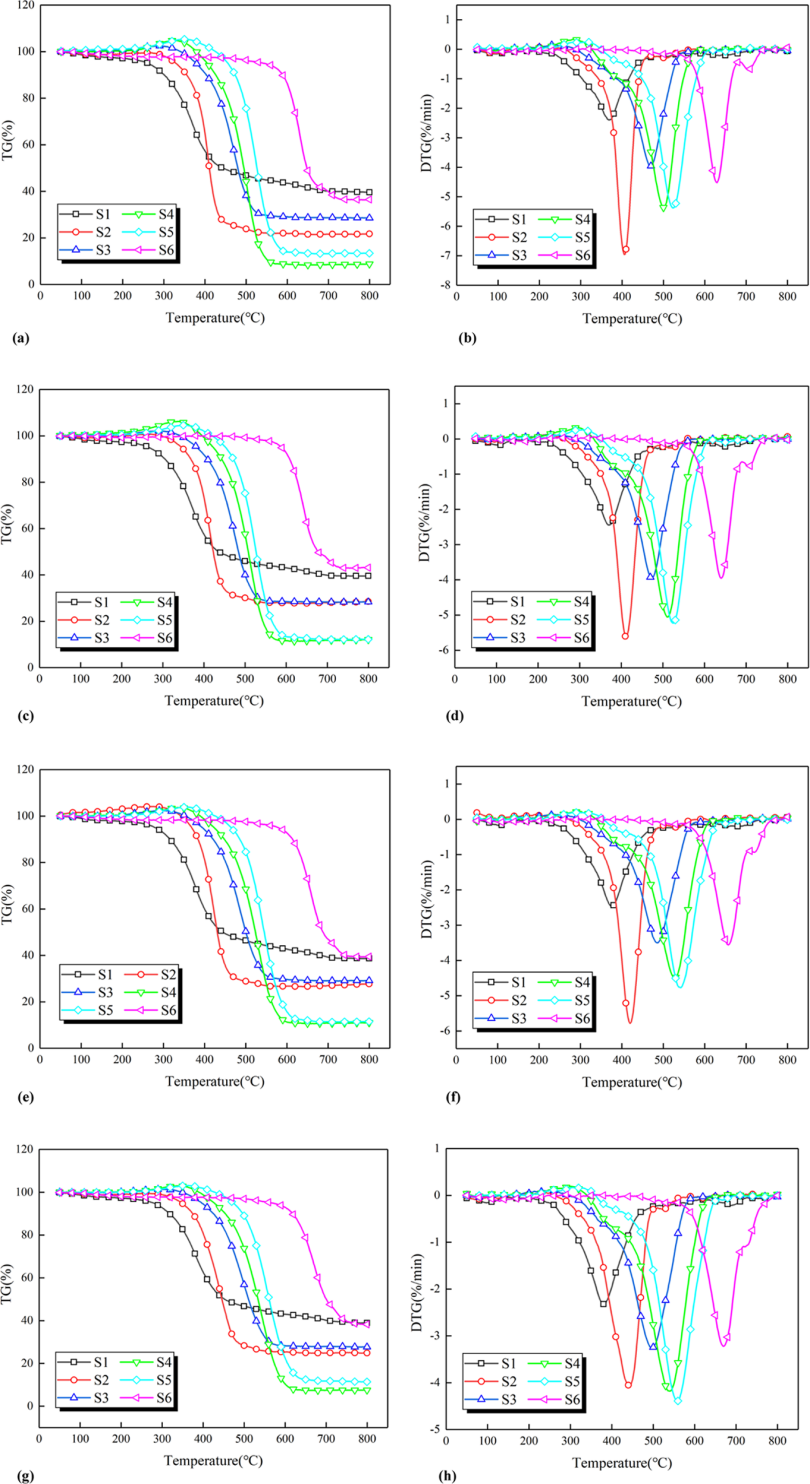
TG–DTG curves of the coal samples. (a) TG curves of the coal samples at an oxygen concentration of 21%. (b) DTG curves of the coal samples at an oxygen concentration of 21%. (c) TG curves of the coal samples at an oxygen concentration of 15%. (d) DTG curves of the coal samples at an oxygen concentration of 15%. (e) TG curves of the coal samples at an oxygen concentration of 10%. (f) DTG curves of the coal samples at an oxygen concentration of 10%. (g) TG curves of the coal samples at an oxygen concentration of 5%. (h) DTG curves of the coal samples at an oxygen concentration of 5%.

The TG and DTG curves for the six samples in different oxygen atmospheres are shown in Figure 1. In the initial stage of CSC, the coal mass increases slightly with the increasing temperature because the gases in the surroundings become physically adsorbed on the coal surface and the physical adsorption is nonselective. At this time, the chemical adsorption on the coal surface is weak, and the coal mass reaches its maximum in a short time.^39^ As the temperature rises, the thermal movement of gas molecules intensifies, and the molecular force cannot be maintained on the coal surface, and then, the gas would be desorbed. Meanwhile, the moisture of coal evaporates and causes a decline in coal mass. With the further increase of temperature, the cyclic macromolecules in coal accelerate the breaking speed, and the movement frequency of functional groups intensifies. Thus, the chemical adsorption capacity of the coal is enhanced and accompanied by the production of carbohydrates. Consequently, the carbohydrate mass is greater than that of the consumed mass of coal, and the coal mass increases once more. Moreover, as the coal reaction keeps accelerating, the adsorption and consumption of oxygen on the coal surface reach an equilibrium, further reducing the coal mass.^40^

As shown in Figure 1, at the same oxygen concentration, the lower coal ranks indicate the lower temperatures required to reach the pyrolysis stage. As the oxygen concentration decreases, the reaction rate of coal decreases. It can be seen that the oxygen concentration influences the process of oxidation and combustion of coal, and the rate of coal reaction is proportional to the oxygen concentration. The reaction processes of different coal samples are similar, but there are still some differences. The samples have their physical and chemical properties, leading to the units with different active structures on the molecule to effectively participate in the adsorption and desorption reactions during the CSC.^41,42^

### 3.2 Critical Temperature

**Figure 2:**
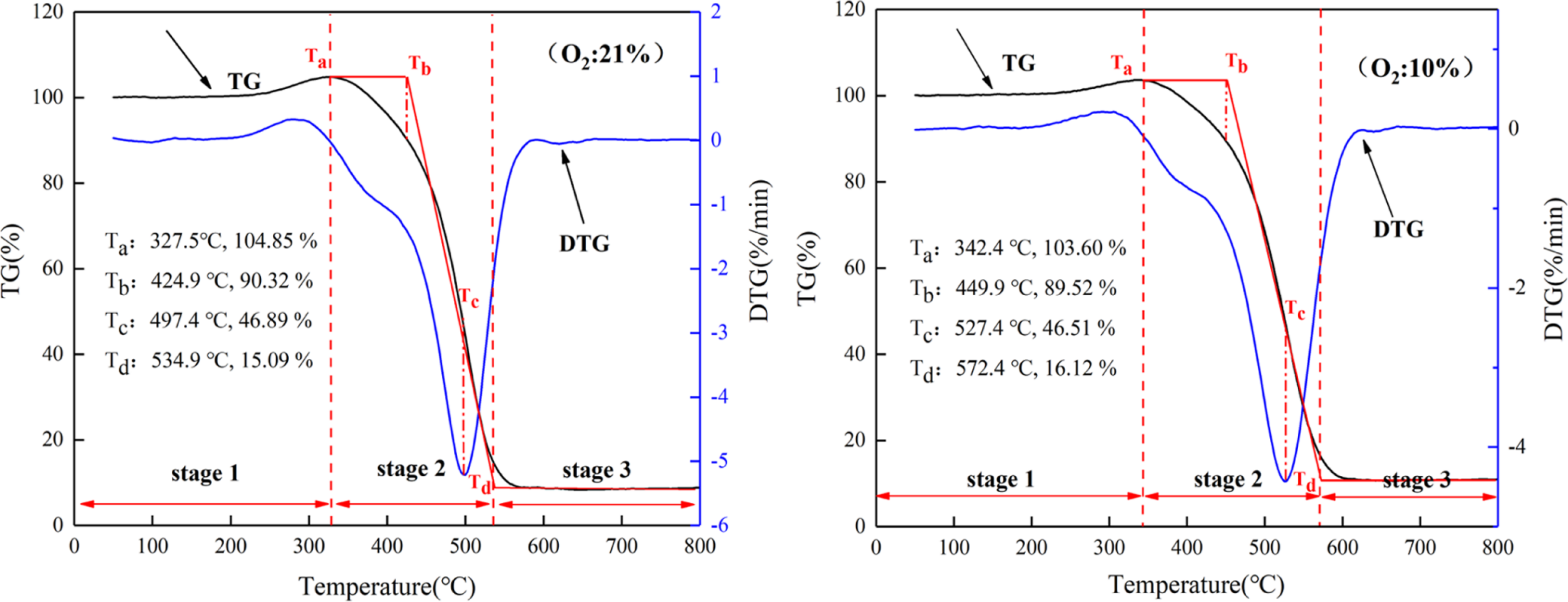
Distribution of critical temperature in S4 at oxygen concentrations of 21 and 10%.

Previous studies have shown that the process of CSC has segmented characteristics. The critical temperature is usually used to distinguish the different reaction stages.^23,30,43^ In the study, the maximum weight temperature (*T*_a_), ignition temperature (*T*_b_), maximum peak temperature (*T*_c_), and exhaustion temperature (*T*_d_) are used to evaluate the oxidation and combustion characteristics of the coals. *T*_a_ is the temperature when the weight of samples by adsorption reaches the maximum values. *T*_b_ is the temperature when the samples begin to burn. *T*_c_, namely, is the point at which the intensity of CSC reaction reaches the highest. *T*_d_ indicates that the combustible materials inside the coal have been burned out. As shown in Figure 2, as the oxygen concentration decreases, the critical temperature in sample S4 increases, indicating that the reduction of oxygen concentration can inhibit the reaction process of CSC.

The critical temperatures of the six coal samples in different oxygen atmospheres are shown in Table 3. The variation of the critical temperature for different samples was almost identical. The lower oxygen concentration indicates a higher critical temperature. In contrast, as the oxygen concentration increases, the active groups in coal are more likely to make contact with oxygen molecules, accelerating the oxidation reaction of coal. As a result, the value of critical temperature decreases. The *T*_a_ of sample S1 and S6 are 289.9 and 592.4 °C, respectively, at an oxygen concentration of 21%. The *T*_d_ are 449.9 and 659.9 °C for sample S1 and S6, respectively. Under the same conditions, it can be seen that the critical temperature of coals is influenced by their properties. S6 is the coal with low reactivity and volatile fraction, causing an overall low critical temperature. Meanwhile, the critical temperatures of sample S1 and S6 decreased when the oxygen concentration was 10 or 5%, but the difference in the critical temperatures was still low.

**Table 3:** Critical Temperature of Six Coals at Different Oxygen Concentrations

		critical temperature (°C)				
atmospheres	coal sample	*T*_a_	*T*_b_	*T*_c_	*T*_d_	weightlessness (%)
21% O2	S1	202.4	289.9	342.4	449.9	60.33
	S2	267.5	362.5	402.4	444.9	78.20
	S3	292.4	397.4	444.9	512.4	71.57
	S4	327.5	424.9	497.4	534.9	91.26
	S5	359.9	474.9	522.4	562.4	86.80
	S6	447.4	592.4	622.4	659.9	63.45
15% O2	S1	184.9	309.9	344.9	437.4	60.25
	S2	274.9	364.9	407.5	450.0	71.33
	S3	294	414.9	449.9	509.9	71.60
	S4	337.5	434.9	489.9	552.4	88.02
	S5	364.9	477.4	524.9	564.9	87.65
	S6	467.4	597.4	637.4	679.9	56.84
10% O2	S1	189.9	319.9	349.9	442.4	61.20
	S2	282.5	392.4	417.5	452.4	72.68
	S3	307.4	412.4	459.9	537.4	70.85
	S4	342.4	442.4	499.9	574.9	89.12
	S5	369.9	494.9	539.9	579.9	88.41
	S6	477.4	604.9	652.4	699.9	60.67
5% O2	S1	214.9	322.4	354.9	452.4	60.96
	S2	284.9	382.5	432.4	479.9	75.05
	S3	314.3	437.4	474.9	539.9	72.38
	S4	347.5	467.4	519.9	584.9	92.42
	S5	374.9	499.9	557.4	604.9	88.54
	S6	484.9	622.4	664.9	714.9	61.57

As shown in Table 3, the coal weightlessness during CSC fluctuated slightly as the oxygen concentration changed. The weight loss of sample S4 and S5 consistently remained above 86.80% as the oxygen concentration varied, while the weight loss of sample S1 and S6 remained in the range of 56.84–61.57%. Because of the lower ash content of sample S4 and S5, leading to the lower mass of solid particles being left over during CSC, which results in a higher weight loss of the sample S1 and S6.

### 3.3 Thermodynamic Model

The process of CSC of each sample was divided into three stages (Figure 2). Stage 1 occurred between *T*_0_ and *T*_a_. Stage 2 occurred between *T*_a_ and *T*_d_. Stage 3 occurred between *T*_d_ and *T*_z_. Some authors have compared different kinetic methods (Friedman, KAS/Miura-Maki, FWO, Kissinger) to highlight the accuracy of calculations of activation energy, but these methods do not have absolute accuracy and the calculation workloads are large.^44,45^ The conversion rates of different coal samples during CSC are different, and their mechanistic function partly determines the difference in activation energy. In other words, the reaction mechanism of different samples during CSC cannot be consistent. Therefore, it is necessary to investigate the mechanism functions of different samples.^30^ In this study, the Malek method is used to infer the most probabilistic mechanism function that describes the process of CSC, and the reliability of this method has been verified by the relevant literature reports.^46^

**Figure 3:**
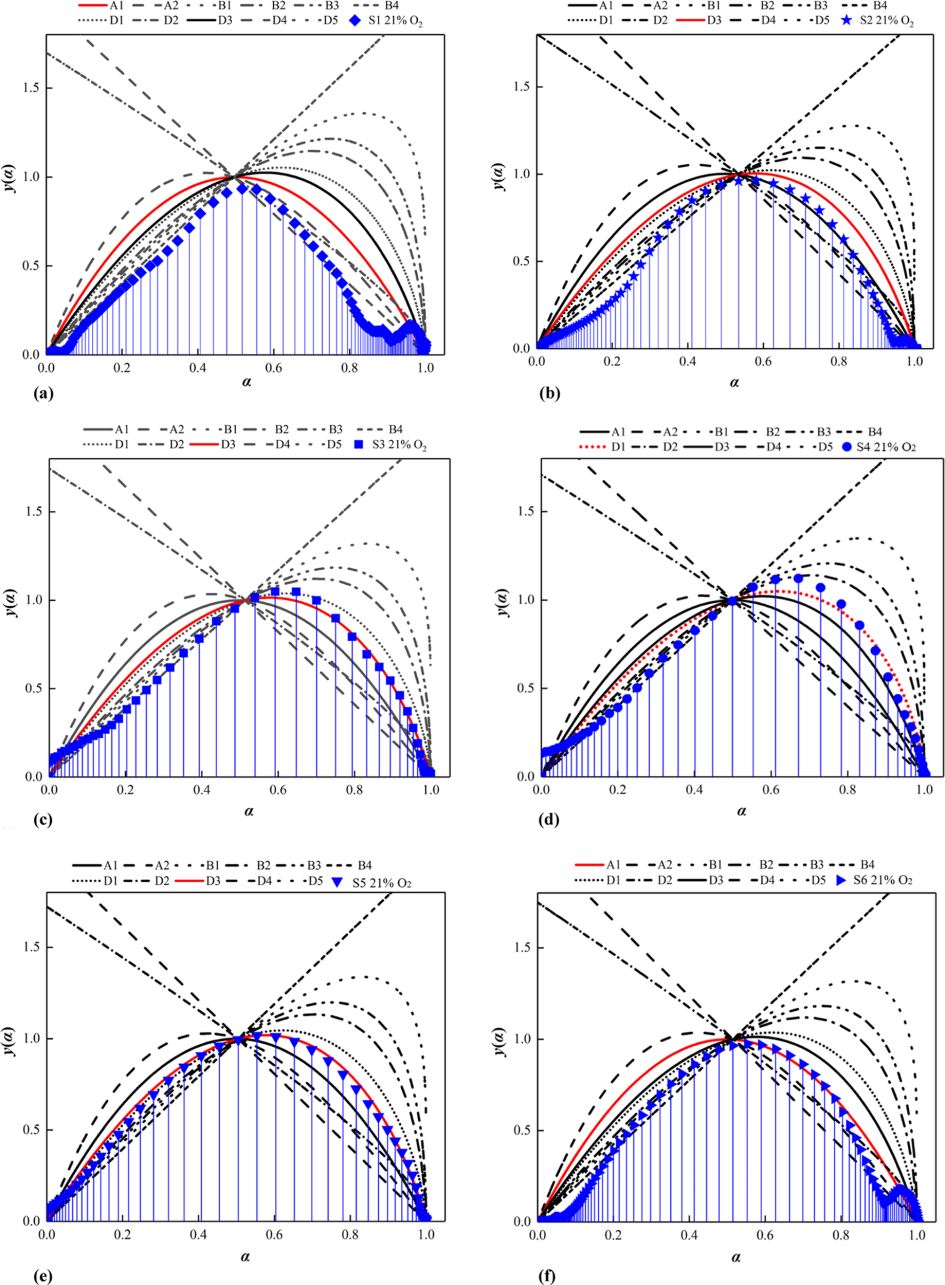
Curves of the most probabilistic mechanism function of the coal samples at an oxygen concentration of 21%. (a) Curve of the most probabilistic mechanism function of sample S1, (b) curve of the most probabilistic mechanism function of sample S2, (c) curve of the most probabilistic mechanism function of sample S3, and (d) curve of the most probabilistic mechanism function of sample S4. (e) Curve of the most probabilistic mechanism function of sample S5 and (f) curve of the most probabilistic mechanism function of sample S6.

Yao^30^ showed that the mechanism function most likely to cause spontaneous combustion of blended coals under enriched oxygen conditions changes after the conversion ratio exceeds 0.55 and suggested that the material mixture may influence the mechanism function. The spontaneous combustion of coal gradually shifted from a homogeneous reaction to an anisotropic reaction, changing the mechanistic function of CSC. However, in this study, raw coal was used for tests under lean-oxygen conditions. We found that the shape of the *y*(α) – α curve for single coal at different oxygen concentrations was almost consistent. When the material properties of coal are unchanged, the oxygen concentration has less influence on the mechanism function most likely to cause CSC. The relationship curves of *y*(α) – α for the six samples at the oxygen concentration of 21% are shown in Figure 3. The mechanism functions of sample S1 and S6 were regarded to be random nucleation and subsequent growth, with the integral function of the kinetic mode (1 – α)^−1^ – 1. The mechanism functions of sample S2, S3, S4, and S5 were considered to be three-dimensional diffusion, with the integral function of the kinetic mode [(1 – α) – 1/3 – 1]^2^ for sample S2, S3, and S5 and the integral function of the kinetic mode [1 – (1 – α)^1/3^]^2^ for sample S4. The same mechanism function was used to calculate the kinetic parameters of the coal sample at different characteristic stages, and the least-squares method was used to fit the kinetic curves segmentally. Finally, activation energies and pre-exponential factors were obtained from the slope and intercept of fitted line of the sample at each characteristic stage.

### 3.4 Relationship between the Mechanistic Function and 1/*T*

**Figure 4:**
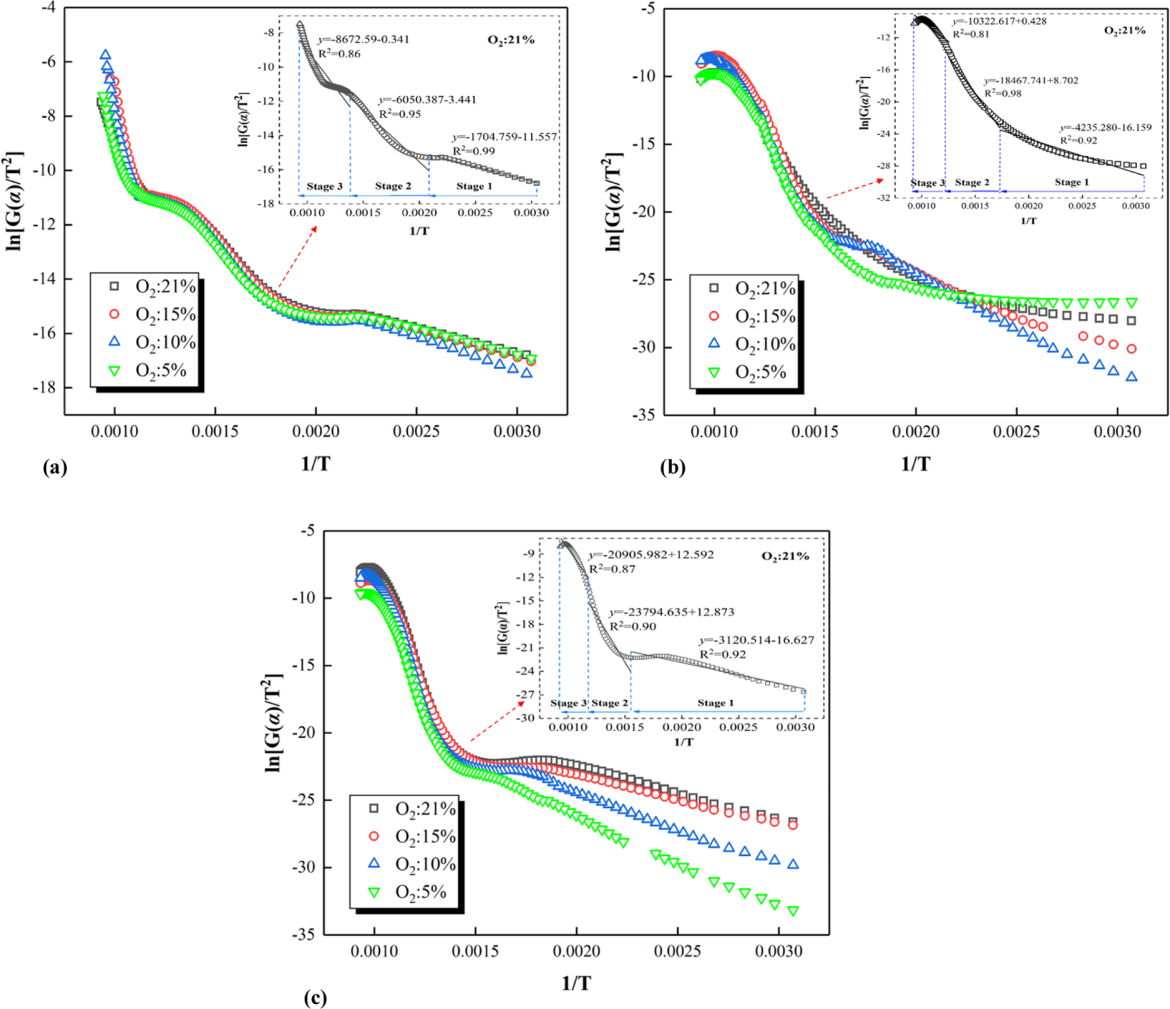
Relationship between the kinetic mechanism function and 1/*T* of different coal samples. (a) Relationship between the kinetic mechanism function and 1/*T* of sample S1. (b) Relationship between the kinetic mechanism function and 1/*T* of sample S3. (c) Relationship between the kinetic mechanism function and 1/*T* of sample S5.

Using eq 4, the scatter diagrams of ln[*G*(α)/T^2^] against 1/*T* of coal samples under lean-oxygen conditions were plotted. The kinetic behaviors for different coal samples (S1, S3, and S5) under lean-oxygen conditions are shown in Figure 4. It can be seen that the scatter values of the coal samples in different oxygen atmospheres show the same trend. At the oxygen concentration of 21%, the scatter values for sample S1 at stage 1 and stage 2 exhibit good linear correlations (*R*_stage1_^2^ = 0.99 and *R*_stage2_^2^ = 0.95), and the stages are also the main stages of CSC. However, the linear correlation (*R*_stage3_^2^ = 0.86) of stage 3 for sample S1 was relatively low because of the small variation in coal mass in stage 3, causing large errors in the calculation. In other words, the discrepancy of linear correlations between the three stages also reflect the inhomogeneity of CSC.^45^ Similarly, the scattered values of samples S3 and S5 at stage 1 and stage 2 also showed good linear correlation (*R*^2^: 0.90–0.98), while their linear correlation (*R*_stage3_^2^ = 0.86) at stage 3 was low. As the oxygen concentration varies, the differences in the slope of relationship curves at stage 1 for different coal samples were greater. Meanwhile, as the oxygen concentration changed, the rank of coals indicated the greater differences of the slope of relationship curves at stage 1. It can be seen that the kinetic behavior of high-rank coals during the process of low-temperature oxidation is more influenced by the oxygen concentration, which is one of the reasons for most scholars to investigate the kinetic characteristics during low-temperature oxidation of CSC.^33−35,47,48^

### 3.5 Thermodynamic Parameters

The thermodynamic parameters of samples during CSC under lean-oxygen conditions are shown in Table 4. From the thermodynamic theory, the activation energy can reflect the difficulty of chemical reaction. The average activation energies of sample S3 at different oxygen concentrations (21% O_2_, 15% O_2_, 10% O_2_, and 5% O_2_) were 92.9, 109.6, 119.9, and 120.7 KJ/mol, respectively, indicating that the increase of oxygen concentration can promote the reaction of CSC. In general, a higher oxygen concentration can accelerate the coal reaction, and it should correspond to lower activation energy.^30^ However, the average activation energy of samples (S4, S5, and S6) increased with the oxygen concentration. The literature^49^ suggests that the activation energy is influenced by the factors such as molecular concentration, the degree of reactive radiation, and organic impurities during CSC. Therefore, when these factors affect the activation energy significantly than the oxygen concentration affects the activation energy, the activation energy may develop in reverse. For the low-rank coals (S1, S2, and S3), the coal reaction was more active with increasing oxygen concentrations. In contrast, for the high-rank coals (S4, S5, and S6), the activation energy remained at a high value even if the oxygen concentration changes. Consequently, the high-rank coals indicate higher activation energy, increasing the difficulty of CSC.

**Table 4:** Thermokinetic Parameters of Coal Samples in Different Atmospheres

		21% O_2_		15% O_2_		10% O_2_		5% O_2_	
samples	stage	*E* (KJ/mol)	*A* (min^–1^)	*E* (KJ/mol)	*A* (min^–1^)	*E* (KJ/mol)	*A* (min^–1^)	*E* (KJ/mol)	*A* (min^–1^)
S1	stage 1	14.2	8.2 × 10^–2^	15.1	1.0 × 10^–1^	18.2	2.5 × 10^–1^	14.0	7.3 × 10^–2^
	stage 2	50.3	9.7 ×10^2^	52.4	1.4 × 10^3^	52.4	1.2×10^3^	47.8	4.0 × 10^2^
	stage 3	72.1	3.1 × 10^4^	86.3	3.7 × 10^5^	93.8	9.6×10^5^	67.8	1.3 × 10^4^
	average value	45.5		51.3		54.8		43.2	
S2	stage 1	36.9	2.8 × 10^–2^	15.7	6.7 × 10^–3^	22.6	2.0 × 10^–3^	8.7	2.2 × 10^–6^
	stage 2	118.0	4.7 × 10^6^	93.7	2.0 × 10^5^	104.5	4.7×10^5^	201.5	1.6 × 10^13^
	stage 3	97.0	8.3 × 10^5^	185.1	1.6 × 10^12^	113.9	5.5×10^6^	99.3	1.8 × 10^6^
	average value	84.0		98.2		80.3		103.1	
S3	stage 1	42.8	2.1 × 10^–2^	50.6	1.5 × 10^–1^	64.8	4.9×10^0^	74.0	5.9 × 10^1^
	stage 2	178.5	3.5 × 10^10^	174.4	1.5 × 10^10^	180.2	3.3×10^10^	201.0	5.6 ×1 0^11^
	stage 3	57.3	1.6 × 10^3^	103.9	3.0 × 10^6^	114.6	9.2×10^6^	87.1	8.3 × 10^4^
	average value	92.9		109.6		119.9		120.7	
S4	stage 1	41.3	6.2 × 10^–3^	22.2	5.5 × 10^–4^	52.3	5.9 × 10^–2^	47.5	1.0 × 10^–2^
	stage 2	132.6	3.0 × 10^6^	126.6	7.6 × 10^5^	143.2	7.5×10^6^	145.4	6.9 × 10^6^
	stage 3	50.6	1.9 × 10^1^	72.0	3.9 × 10^2^	75.4	5.3×10^2^	93.2	4.9 × 10^3^
	average value	74.8		73.6		90.3		95.4	
S5	stage 1	25.9	9.3 × 10^–4^	25.9	5.5 × 10^–4^	43.0	1.8 × 10^–2^	57.4	1.5 × 10^–1^
	stage 2	197.8	4.7 × 10^10^	200.9	5.4 × 10^10^	204.1	5.1×10^10^	216.0	2.1 × 10^11^
	stage 3	173.8	2.5 × 10^5^	156.0	1.6 × 10^9^	175.4	2.0×10^10^	154.5	3.7 × 10^8^
	average value	132.5		127.6		140.8		142.6	
S6	stage 1	16.5	7.0 × 10^–2^	6.6	4.2 × 10^–4^	12.8	1.9 × 10^–2^	20.7	2.3 × 10^–1^
	stage 2	111.8	1.9 × 10^5^	232.5	2.9 × 10^12^	138.4	5.5×10^6^	125.7	7.2 × 10^5^
	stage 3	412.1	7.6 × 10^22^	272.5	2.3 × 10^15^	505.5	2.1×10^27^	461.3	2.6 × 10^24^
	average value	180.1		170.5		218.9		202.6	

**Figure 5:**
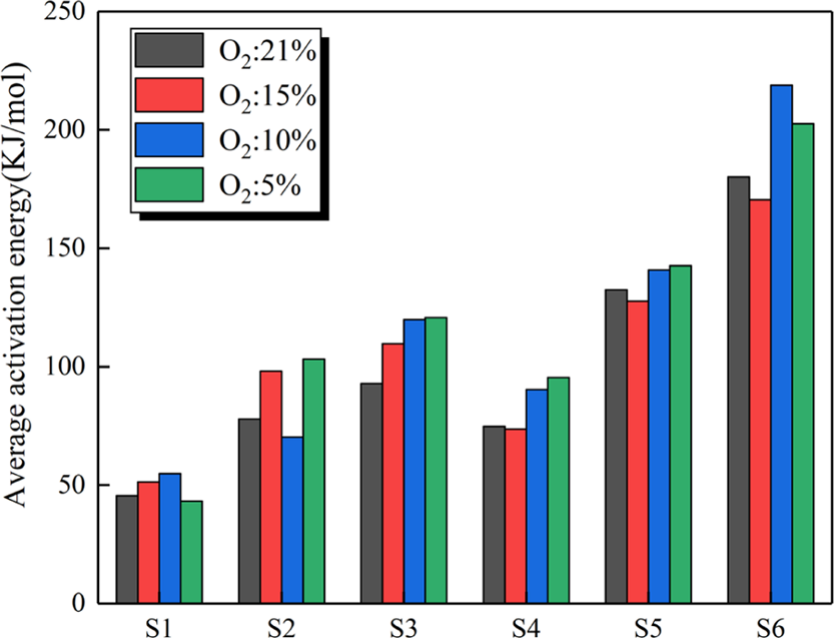
Average activation energies of coal samples at different oxygen concentrations.

As shown in Figure 5, the average activation energy of coal samples increases with decreasing oxygen concentration. It can be seen that the effect of oxygen concentration on the average activation energy was small. Under lean-oxygen conditions, the ranks of coal have a much more significant effect on thermodynamic parameters than the oxygen concentrations.

### 3.6 Kinetic Compensation Effect

**Figure 6:**
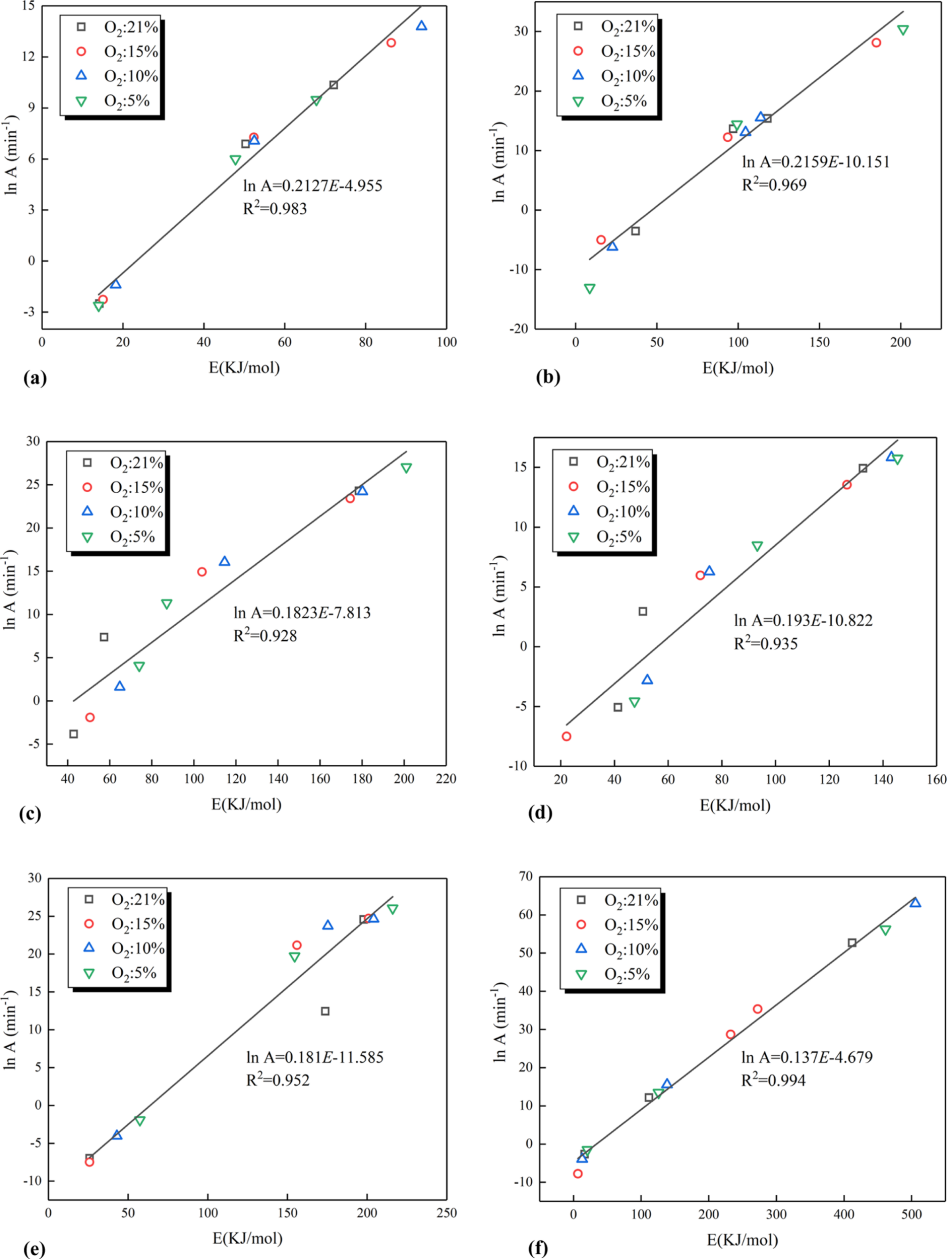
Linear relationship between In *A* and *E* of coal samples at different oxygen concentrations. (a) Linear relationship between In *A* and *E* of sample S1 (b) Linear relationship between In *A* and *E* of sample S2. (c) Linear relationship between In *A* and *E* of sample S3. (d) Linear relationship between In *A* and *E* of sample S4. (e) Linear relationship between In *A* and *E* of sample S5. (f) Linear relationship between In *A* and *E* of sample S6.

Based on the transition state theory, Ding et al.^50^ investigated the kinetic effect of the gasification reaction between coal and water. They suggested that the unstable intermediate transition state during the gasification process contributed to the formation of a compensation effect, with lower activation energy being required for the reaction of active coal molecules and water molecules. Furthermore, the activation entropy required to form activation complexes with water molecules on the coal surface was also reduced. Thus, the pre-exponential factor decreases with decreasing activation energy. Likewise, there is an intermediate transition stage in the combination of coal molecules and oxygen molecules.^22,27^ Consequently, the kinetic compensation effect of CSC under lean-oxygen conditions can also be explained quantitatively by transition state theory. The linear relationship between In *A* and *E* of six coal samples at different oxygen concentrations are shown in Figure 6. The In *A* and *E* values of coal samples exhibited a good linear correlation (*R*^2^: 0.93–0.99), indicating a kinetic compensation effect for the oxidation and combustion of coal under lean-oxygen conditions. The pre-exponential factor showed a positive correlation with the activation energy. The pre-exponential factor represents the effective collision frequency of molecules during CSC. Therefore, the frequency of effective intermolecular collisions increases with increasing values of the pre-exponential factor, raising the difficulty of CSC. As a result, the activation energy increases.^32,44^ However, most authors often overlook the influence of errors on experimental results in existing studies involving kinetic compensation effects. Indeed, many literature reports have detailed the experimental precision and error, but most of the them are based on a single experimental variable, leading to uncertainty in the conclusions. Furthermore, two of the most important conditions for the study of kinetic compensation effect of inhomogeneous reactions are^51^ (1) a suitable kinetic model and (2) suitable experimental variables. Based on this idea, we have investigated the kinetic compensation effect during CSC from multiple perspectives, including the lean-oxygen conditions and the rank of coal. This is of positive significance to guide the study of thermodynamic properties of CSC.

## 4 Conclusions

An investigation was undertaken to understand the effect of coal ranks on the thermodynamic behavior during coal oxidation and combustion under lean-oxygen conditions. Based on TG analysis, the CSC process was divided into three stages and described with critical values. The results showed that the coal ranks have a negative effect on coal oxidation and combustion under lean-oxygen conditions. The difference in the critical temperature of different coals was small as the oxygen concentration varied. On the other hand, the oxygen concentration has a small effect on the thermodynamic behaviors, while the coal rank has a greater influence on it. The analysis of the kinetic model and parameters approved this conclusion as well. Under lean-oxygen conditions, the oxygen concentration has less effect on the mechanism function most likely to describe the thermal oxidation process of coal. Meanwhile, there was a kinetic compensation effect during the CSC under the lean-oxygen atmosphere.
